# Brain Tumor at Diagnosis: From Cognition and Behavior to Quality of Life

**DOI:** 10.3390/diagnostics13030541

**Published:** 2023-02-02

**Authors:** Daniela Pia Rosaria Chieffo, Federica Lino, Daniele Ferrarese, Daniela Belella, Giuseppe Maria Della Pepa, Francesco Doglietto

**Affiliations:** 1 Clinical Psychology Unit, Fondazione Policlinico Universitario Agostino Gemelli IRCCS, 00168 Rome, Italy; 2 Department Women Children and Public Health, Catholic University of Sacred Heart, 00168 Rome, Italy; 3 Department of Neurosurgery, Fondazione Policlinico Universitario Agostino Gemelli IRCCS, 00168 Rome, Italy; 4 Department of Ageing, Neurosciences Head Neck and Orthopedics Sciences, Catholic University of Sacred Heart, 00168 Rome, Italy

**Keywords:** behavioral symptoms, brain tumor, diagnosis, cognitive symptoms, effective communication, glioma, meningioma, pituitary adenoma, quality of life

## Abstract

Background: The present narrative review aims to discuss cognitive–emotional–behavioral symptoms in adults with brain tumors at the time of diagnosis. Methods: The PubMed database was searched considering glioma, pituitary adenoma, and meningioma in adulthood as pathologies, together with cognitive, neuropsychological, or behavioral aspects. Results: Although a significant number of studies describe cognitive impairment after surgery or treatment in adults with brain tumors, only few focus on cognitive–emotional–behavioral symptoms at diagnosis. Furthermore, the importance of an effective communication and its impact on patients’ quality of life and compliance with treatment are seldom discussed. Conclusions: Adults with brain tumors have needs in terms of cognitive–emotional–behavioral features that are detectable at the time of diagnosis; more research is needed to identify effective communication protocols in order to allow a higher perceived quality of life in these patients.

## 1. Introduction

Primary brain tumors represent 1.6% of all cancers [1]. Patients with primary and secondary brain tumors often present multiple symptoms such as motor deficits, emotional or cognitive impairment, headache, or somatic changes. Treatment of brain tumors can, in some cases, induce additional cognitive symptoms.

Although cognitive and emotional–behavioral disorders represent a challenge in the diagnosis and treatment of brain tumors, there are no confirmed epidemiological data on the prevalence of these disorders in patients with brain tumors at the time of diagnosis. In particular, in the literature, there are only fragmented and non-homogeneous data on these disorders, mostly after treatment—as a matter of fact, the real burden of cognitive–behavioral changes in the neurooncological patients is still largely unknown.

Cognitive problems at diagnosis can have a large impact on patients’ quality of life, particularly as many patients are at a young age at diagnosis (with implications for family, work, and social life). Some studies have investigated the link between cognition and quality of life in patients with brain tumors and some others explored the implications of effective diagnosis communication in brain tumor patients. 

The present review aims to describe cognitive symptoms in patients with brain tumors at the time of diagnosis and association with perceived quality of life and effective communication.

## 2. Materials and Methods

The PubMed database was searched with the combination of the following terms: “adult brain tumor”; “glioma”; “adenoma”; “meningioma”; “neuro-cognitive symptoms”; “neuropsychological effect”; “neuro-behavioral”; “affective symptoms”; “mood in brain tumor”; “behavioral symptoms”; “brain tumor”; “preoperative symptoms in brain tumor”; “preoperative signs”; “communication”; “cancer”. Inclusion criteria were articles published in English. Single case studies were excluded. Articles were screened on the basis of their abstract and selected ones were read in their entirety.

## 3. Symptoms at Diagnosis

Signs and symptoms of brain tumors are usually described as general or focal. Generally, a low-grade tumor corresponds to focal signs that generalize with the increase in grade or dimension of the tumor. General symptoms usually include vomiting, nausea, headache, sensory deficits, and impaired cognitive and emotional functions [2]. Cognitive deficits are often present at the time of diagnosis. Neurocognitive function is of great importance in patients with brain tumors. Even patients in good neurological condition may suffer from some neurocognitive dysfunction affecting their daily living. Cognitive decline is a reliable indicator of brain tumor progression, even before this becomes evident on brain imaging studies [3]. For this reason, pre- and post-treatment neuropsychological assessments are of great importance from a clinical perspective. Preoperative neuropsychological evaluation also helps to plan rehabilitation and gain social adjustment. Whereas the success of brain tumor surgery is traditionally measured by the extent of tumor resection and survival, neurocognitive dysfunction has emerged as another important outcome measure. Neurocognitive testing in newly diagnosed brain tumor patients often elucidates deficits in particular cognitive tasks. These may have an impact on social life, professional life, and of course, therapy decisions. For these reasons, awareness of cognitive deficits is of great relevance for the management of the patient from the very beginning of his or her treatment pathway. 

From a cognitive–behavioral perspective, while neuropsychological data can accurately describe the clinical picture in patients with brain tumors, accurate analysis of emotional symptoms can further help to better define diagnosis. 

Symptoms such as apathy or anhedonia are, in fact, often misinterpreted as mood deflection or depression, but it is now clear that they are often a neurological sign. 

Nonmedical factors (intended as characteristics different from medical and surgical ones) are rarely studied as predictors of surgical outcome. They include variables such as family situation, socioeconomic status, personal characteristics, relationships, and social support.

In a recent review by Schiavolin and colleagues, depressive symptoms, altered mental status, personality traits, and autonomy for daily activity were found to be significant psychological outcome predictors [4]. In patients with meningioma, preoperative depressive symptoms were found to be predictors of shortened survival in meningioma, while conflicting results exist in the literature regarding their impact in glioma patients [5].

Other signs and symptoms, such as fatigue, can significantly affect patients as they directly impact their quality of life. Fatigue can be defined as a multidimensional construct [6] which underlies a multiple (mental and physical)-symptom concept. It describes a state where it is difficult to initiate and sustain voluntary activities while maintaining an adequate alertness to process sensory information. It may represent a clinical symptom of a disease if it occurs with insufficient provocation as it happens in brain tumor patients at the pre-surgical stage. The percentage of patients suffering from fatigue fluctuates between 34% and 43% in meningioma patients [7] and reaches up to 82% of low-grade glioma patients [8,9]. Fatigue is today mostly measured by self-report questionnaires. For this reason, De Dreu and colleagues designed a study to search for a neuropsychological correlate for fatigue in brain tumor patients, finding consistent results between various brain tumor sub-groups, despite demographical and pathological differences. They found an association between fatigue and objective measurements of brain activity, specifically the default mode network activity related to phasic alertness. This finding could increase the reliability of fatigue assessment in brain tumor patients and help in studying the underlying neurophysiology.

## 4. Meningioma

Meningiomas are central nervous system tumors most frequently discovered in middle to late adult life. Even if the tumor does not grow from brain tissue but emerges from meninges, it is commonly referred to as a brain tumor. Around 90% of meningiomas are benign, while a small percentage of them are atypical and malignant. A large percentage of patients in whom a meningioma is diagnosed undergo surgery with complete resection, which is often curative. 

Meningiomas are usually slow-growing tumors with an insidious onset of symptoms [10]. They may present several focal neurological symptoms or neuropsychological deficits depending on their location. The most common sites of origin are the skull base and convexity, and sites of dural reflections [11]. 

Neuropsychological symptoms are often present at diagnosis and the most affected domains are memory, attention, and executive functions. Tucha and colleagues [12] documented significant pre-operative impairment in working memory, attention, and executive function in patients with frontal meningiomas. Deficits in memory and attention show significant improvement after surgery. Meskal and colleagues [13] studied a sample of 68 meningioma patients and documented lower scores in pre- and post-operative assessments compared to American normative data for reaction time, cognitive flexibility, attention, memory, and psychomotor speed. Very low scores were documented in 69% of patients in at least one cognitive domain, with significant improvement after surgery. Other studies have confirmed abnormal results in neuropsychological testing before surgery [14,15]. In general, studies confirm that meningioma patients have a neuropsychological profile in which memory, attention, and executive functions are most affected. Surgery generally has a beneficial, even if not decisive, impact on cognitive functioning. 

Psychological distress and cognitive functioning deficits also affect patients’ quality of life. Psychological distress in the pre-operative period has been related to worse cognitive abilities more than objective neuropsychological performance. 

Anterior skull base meningiomas often involve the ventromedial prefrontal cortex, which is implicated in various higher cognitive and behavioral functions. Abel and colleagues [16] documented deficits in adaptive functions and real-life decision making in these patients through the Iowa Gambling Task (IGT), which evaluates and quantifies decision making deficits during conditions of immediate and delayed reward and punishment. Moreover, the study demonstrated a significant decline in adaptive function from pre- to post-surgery, finding an association between IGT and adaptive behavior; it is possible that at least some of the adaptive function impairment occurs secondary to impaired decision making [17].

Subjective cognitive functioning (SCF) has also been explored by Van Lonkhuizen and colleagues [18] through the Cognitive Failures Questionnaire (CFQ) [19], comparing SCF with normative data. In contrast with previous studies, meningioma patients reported better SCF compared with normative controls preoperatively and 3 months after surgery. Most patients reported high SCF at all time points. This result is impressive given the high percentages of brain tumor patients with cognitive complaints found in previous studies. The authors explain this finding with the fact that during recovery from surgery, patients are (partly) disburdened from their daily roles and responsibilities and this may result in limited experience of cognitive complaints. In addition, when faced with changes in health status, patients might alter their internal standards and values and also SCF might be reconsidered in regard to changes in health status.

A systematic review by Zamanipoor Najafabadi and colleagues [20] explored health-related quality of life (HRQoL) in meningioma patients. This construct, already investigated in the 90s by Guyatt and colleagues, is a multidimensional concept covering several aspects of a patient’s life including disease and treatment [21] and can be viewed as a latent construct which describes several domains: physical, role functioning, social, and psychological aspects of well-being and functioning. In addition, in contrast to QoL, HRQoL can include both objective and subjective perspectives in each domain.

In general, meningioma patients report worse HRQoL levels compared with healthy controls before surgery, though not all studies are homogenous. Global HRQoL improved significantly after surgery in long-term evaluations (10–58 months). This study also compared meningioma and glioma patients: the former reported better HRQoL score for several dimensions, such as cognitive, social, and physical functioning. Factors influencing HRQoL were tumor size, histological grade, and tumor recurrence. Long-term follow-up showed persistent reduced HRQoL in meningioma patients compared with healthy controls. These results suggest an impaired HRQoL even years after anti-tumor treatment in this population: meningioma patients scores are similar to those of a chronic disease.

## 5. Glioma

Malignant gliomas are the most frequent primary central nervous system (CNS) tumor. It is possible to divide gliomas into low grade (slowly progressing) and high grade (fast progressing). The former are usually symptomatic when relatively diffuse, since symptoms occur slowly and insidiously; the latter have poor prognosis [22].

Acevedo-Vergara and colleagues conducted a systematic review on cognitive deficits in high-grade glioma patients [23]. The rapid progression of high-grade tumors is associated with reduced brain plasticity [24]. It is thus intuitive that high-grade gliomas produce diffuse cognitive impairment affecting executive function, memory, attention, and language depending on the location of the tumor. A deficit in the attention domain is usually related to a glioma in the frontal areas, thalamus, anterior cingulate, and parietal cortex [25,26]; deficits in the memory domain may occur in the case of gliomas in the dominant dorsolateral areas of the frontal lobe, dominant temporo-parietal areas, basal ganglia, and hippocampal areas.

Language deficits are usually related to Broca’s, Wernicke’s, and medial frontal gyrus areas. Gliomas infiltrating specific white matter fibers (uncinate and arcuate fasciculus) can also affect language [27]. Executive function deterioration is usually related to frontal areas, but infiltrating tumors can affect white matter fibers and lead to generating dysexecutive syndromes.

Brain tumors in the cerebellum, particularly located in the right cerebellar hemisphere, can be the cause of Cognitive Cerebellar Affective Syndrome (CACS) [28,29]. CACS, first described by Schmahmann and Sherman in 1998 [30], is characterized by disturbances in executive function, impaired visuospatial skills, personality change, and linguistic deficit, resulting in general cognitive impairment.

In light of the general cognitive impairment, prognosis, and implications on daily life, gliomas easily have a negative impact on patients’ quality of life. Sagberg and colleagues [31] investigated the impact of tumor location on HRQoL and concluded that patients with brain tumors in motor-related regions have a poorer HRQoL. The authors explain the data by indicating that these areas must be crucial from the patients’ perspective.

As previously mentioned, Guyatt and colleagues found that HRQoL is worse in gliomas than in meningiomas patients. Emotional disturbances are often present in glioma patients. A systematic review by Mugge and colleagues defined the complex interconnection between depression and glioblastoma, highlighting that complicated concomitant conditions warrant attention when it comes to diagnosis and treatment [32]. Depression and glioblastomas share intricate networks of connectivity and this must be considered in pharmacotherapy. In addition, over time, depression can persist after cancer treatment, affecting the patient’s quality of life in the long term. Preoperative depressive symptoms have been found to be predictors of shorter survival in high-grade glioma (even if no consensus in the literature has been found), as well as in meningioma. No correlation has been found between anxiety and survival in either group and no personality trait was related to survival in glioma patients [33].

Among emotional–behavioral symptoms in patients with glioma, fatigue is frequent. Fatigue is probably linked with the loss of quality of life that glioma patients often experience and with depressive symptoms and sleep disturbance. Cancer-related fatigue (CRF) is one of the most commonly reported cognitive–behavioral symptoms in glioma patients. It is “a distressing, persistent, subjective sense of physical, emotional, and/or cognitive tiredness or exhaustion related to cancer that is not proportional to recent activity and interferes with usual functioning” [34]. It affects up to 96% of patients at different stages of the disease [35]. A qualitative study refers to it as the most severe of patients’ symptoms [36]. Others have documented with fMRI an association of the multidimensional fatigue inventory (MFI-20) scores with brain activity documented in the central executive network [37], suggesting a potential biomarker for CRF in both meningioma and glioma patients. Since survival in glioma patients is poor, with high symptom burden, a substantial part of care should address the quality of survivorship. In light of this conclusion, Röttgering and colleagues [38] are carrying out a randomized controlled trial implementing cognitive behavioral therapy for glioma patients, aimed at fatigue-maintaining beliefs and behavior. 

The incidence of preoperative behavioral symptoms has not been described in detail [39]. Major syndromes such as depressive or anxiety disorders have been described in glioma patients, but more research is needed to evaluate the behavioral profile in glioma patients.

## 6. Pituitary Adenoma

Pituitary adenomas (PA) represent 10–15% of all intracranial tumors [40]. They can be classified as functioning, which cause hormone hypersecretion, and non-functioning, which usually cause a mass effect and/or hypopituitarism. Other than medication and radiotherapy, the main treatment for PA is surgery. Today, the vast majority of patients are treated with the endonasal transsphenoid-sella approach, widely used in clinical treatment, avoiding adverse effects of craniotomy and minimizing the damage to surrounding brain tissues. 

Neurocognitive impairment has been reported by Hendrix [41], as well as Tiemensma and colleagues found reduced quality of life (QoL) [42] and a higher prevalence of psychological disturbances in these patients. 

A systematic review by Pertichetti and colleagues [43] explored the neuropsychological status in patients with PA, patients’ perceived QoL, and the presence of associated psychiatric symptomatology. Cognitive deficits, especially in memory and executive functions, have been reported in patients with PA. At pre-surgical time, may factors could contribute to these deficits, such as hormonal imbalance, malfunction of neuroanatomical structures needed for normal memory processing, or tumor mass effect.

Wang and colleagues [44] reported a high incidence of pre-surgical cognitive dysfunction and post-operative improvement. They attributed amelioration to the improved post-operative endocrine condition, hypothesizing a specific hormone-related cognitive deficit. 

Hendrix and colleagues investigated the psychological effects of suprasellar extension in PA and documented not only cognitive impairment, but also psychopathological disturbances ]. They attributed the finding to the effects of previous hormonal excess on the central nervous system, which might have long-lasting effects. 

Among hypersecretion syndromes, Cushing disease (CD), which is caused by overproduction of adrenocorticotrophic hormone (ACTH) leading to high cortisol levels, is frequently associated with cognitive [45] and psychological disturbances. Among cognitive deficits, the memory domain is most affected, but language, reasoning, and visuospatial performance might also be affected. Emotional disturbances, apathy, melancholia, depressive or anxious symptoms, and impulsivity have been described in these patients. Emotional symptoms could remain with increased prevalence even after long-term remission. Depressive disorder is evident in 50–80% of cases [46]. Studies in the literature on CD showed that even when patients meet the criteria for biochemical remission after surgery, it was associated in the smallest improvement in QoL measurements compared to patients with other PA. Researchers substantiated these findings considering the permanence of the comorbidities and the long exposure to the hormonal secretion [47].

Prolactin secerning pituitary adenomas (prolactinoma) show a distinct personality profile compared to other adenomas: they seem to be more neurotic, depressed, and stressed [48]. This result is potentially related to pituitary lesion or associated with hormonal dysregulations and comorbidities. In addition, acromegaly (caused by prolonged overproduction of GH) is associated with reduced impulsivity and novelty-seeking behavior, which can affect patient QoL.

After surgery, cognitive–behavioral symptoms might not normalize [49] and long-term quality of life remains decreased [50]. 

**Figure 1:**
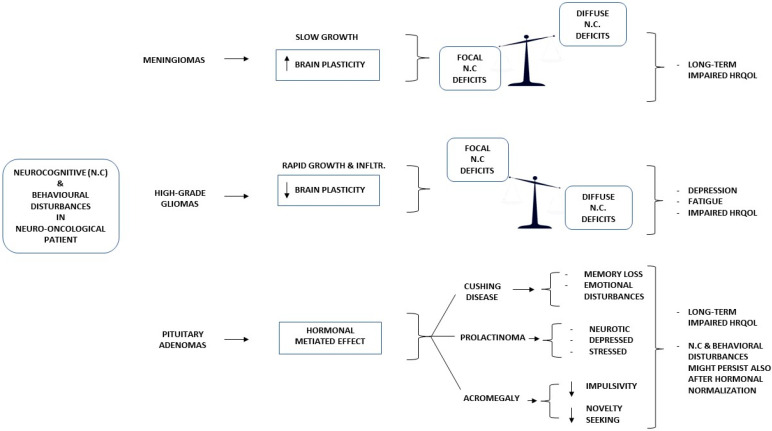
Neurocognitive and behavioral disturbances in relation to emotional distress and quality of life measures.

Figure 1 gives a synthesis of neurocognitive and behavioral disturbances in relation to health-related quality of life measures and emotional disturbances in patients with meningiomas, high-grade gliomas, and pituitary adenomas.

## 7. Communication of Diagnosis

Most studies concentrate on outcome measures and therapies for brain tumors, and only a few shed light on what happens before and at the time of diagnosis. Very little has been discussed, for example, on the communication of diagnosis for patients with brain tumors. Most of the literature focusing on diagnosis deals with the topic of diagnostic procedures and good practices, but very little space is left to the effects of communication.

This is counterintuitive if we consider how significantly the communication of diagnosis can affect the patient’s motivation for therapy and deployment of coping resources. The literature widely underlines the meaning and impact of interpersonal communication in cancer prevention and control. Effective communication is essential in every phase of the disease, including the communication of diagnosis and prognosis, informed choice of therapeutic pathways, and end-of-life care. The literature shows that patient–practitioner communication begins long before receiving a diagnosis. In fact, this connection would already take place during cancer prevention campaigns, having a direct effect on some behaviors aimed at health promotion (such as smoke cessation or screening testing for some types of tumors). When it comes to doctor–patient communication, we can not only consider the amount of information conveyed but “how” the information is disclosed to the patient. This qualitative detail assumes crucial importance in clinical communication. 

This is particularly noticeable in some studies which have shown how participatory communication (involving the patient in decision making and allowing him or her to ask questions) has a direct impact on the probability that the patient undertakes a cancer screening procedure [51,52,53]. 

Among the qualitative impacts of patient–practitioner communication, “health literacy” has been highlighted, which is defined as the ability to access, understand, and communicate health-related information [54,55,56,57]. “Health literacy” is an umbrella term considering different aspects of patients’ interaction with the health system: from the ability to communicate, to the ability of being involved in clinical care and to access health care. Hence, health literacy is an independent risk factor for poor health outcome to be considered when we talk about effective communication. In their review, Koay and colleagues cite several studies demonstrating that health literacy deeply impacts a patient’s understanding of their diagnosis, prognosis, and therapeutic possibilities [58,59]. 

In the literature, a recent increase in the interest of researchers in the field of health literacy is present. However, much of this research does not specifically address patients with brain tumors. It would be valuable if studies targeting this type of population were carried out to explore any “health literacy issue” prevalence in patients with brain tumors and their caregivers [60,61,62].

When it comes to cancer diagnosis, patient–provider communication commonly includes discussions of diagnostic and prognostic information, treatment options, possible side effects, potential psychosocial concerns, and in some cases, palliative care and end-of-life issues. The moment of diagnosis is therefore a delicate moment in which the patient receives a huge series of decisive information. Chawla and colleagues demonstrate gaps in quality of communication (described as a lack of detailed communication regarding late or long-term effects, lifestyle recommendations, or emotional and social needs) experienced by a number of cancer patients at this stage [63]. 

Communicating a diagnosis is an extremely delicate procedure and clinicians are facing a major challenge: they must preserve their patients’ hope while at the same time giving them accurate information. On the other hand, the patient faces a great challenge: processing new information about his or her health while experiencing emotional turmoil. In 2011, Lobb and colleagues explored in their study patients’ and caregivers’ perceptions of communication and prognosis in high-grade glioma patients. They found that clinicians should consider different implications for communication in low-stress versus high-stress diagnosis settings (such as post-surgical intensive care settings), and a requirement for information about prognosis to be tailored to the individuals’ coping abilities has emerged [64,65]. 

When it comes to effective communication in brain tumor patients, as previously discussed, a barrier could be represented by cognitive deficits. Some brain tumors could interfere with cognitive functioning already at the time of diagnosis. Patients could experience difficulties in speaking, language comprehension, attention, abstract reasoning, or many others. Such difficulties could contribute to a lack of awareness. Evidence in the literature indicates that before a brain tumor diagnosis, 24.9% of patients show some mental status change [66]. Therefore, an adjustment in information delivery should be considered by physicians in order to adapt information to patients’ cognitive abilities.

In 2018, in order to reduce the communication gap and enhance shared decision making in patients with glioma, Van De Belt and colleagues [67] elaborated a tailored 3D-printed model of the brain based on patients’ neuroimaging studies. 

This three-dimensional model including surrounding functional areas acted as a communication facilitator, stimulating the patient to ask questions based on the model and improving patients’ understanding about their situation and treatment options. Although 3D models are increasingly used in surgical planning, less attention has been paid to their use in patient education, but they are proving to be promising and simple tools to use.

Some studies have shown that effective communication between patients and physicians favors treatment compliance, patient satisfaction, and promotes detection of reactive psychological symptoms [68,69,70]. 

Ineffective communication leads to insufficient detection of psychological disturbances. Some interesting studies focused on the communication of brain tumor diagnosis to children. Researchers observed that internalizing symptoms is related to ineffective communication. Historically, there has been a shift from a “protective” (or “never tell”) approach to disclosure during the 1950s to an “always tell” approach around the 1980s. Today, particular attention is paid to tailoring the contents of the disclosure based on the developmental stage the child is going through.

In recent literature, diagnostic communication in children, when effective (described as complete, truthful, consistent, comprehensible, gradual, continuous, and tailored), is related to better outcomes [71]. A significant relationship between the onset of internalizing problems, withdrawal, anxiety and depression, social problems, and ineffective communication about the disease has been observed in children [72,73]. More research is needed to determine significant variables and accurate tools to detect the onset of reactive symptoms in adulthood after diagnosis communication. 

Although communication skills are core skills in medicine, only few papers in the literature have accurately detailed communication skills and training programs or guidelines for physicians [74,75]

The American Society of Clinical Oncology Educational Book in 2018 proposed a guideline developed by a multidisciplinary, multiprofessional panel to address this need. The guideline, based on a systematic review of the literature, is structured around nine key areas: communication skills, discussing goals of care and prognosis, discussing treatment options and clinical trials, discussing end-of-life care, using communication to facilitate family involvement in care, meeting the needs of underserved populations, communicating effectively when there are barriers to communication, discussing cost of care, and clinician training in communication skills.

An integration of standardized stress management skills through proper training on communication skills in the physician’s curriculum could significantly help patients in achieving the best possible diagnostic communication and best possible quality of life.

## 8. Conclusions

Brain tumors are associated with cognitive, emotional, and behavioral symptoms. Depending on the tumor’s location, the neuro-behavioral profile changes. These issues are detectable at the time of diagnosis and generally undergo evolution following tumor progression and surgical, pharmacological, and/or radiotherapy treatment. In the literature, there are only fragmented and non-homogeneous data on these disorders, as well as brain metastases [76]. A better understanding of the epidemiology of these symptoms will help identify individuals who are at the greatest risk of developing them and guide clinicians in selecting therapeutic pathways [77,78,79].

Quality of life in brain tumor patients is challenged by the integrity of functional autonomy and by the presence of non-medical factors, such as psychological syndromes, coping strategies, and social support. Cognitive performance also plays a significant part in this challenge. 

Quality of life influences compliance with treatment, which is also affected by the quality of diagnostic communication. It is intuitive, given the present discussion, that ineffective communication leads both to reduced detection of the patient’s symptoms and to a reduction in treatment compliance, as a secondary effect. The existing body of literature considering preoperative cognitive status in relation to QoL and patient–practitioner communication is sparse. Further studies are needed in the future to shed light on the composite interrelation between cognitive symptoms, effective communication, and perceived quality of life. Research in brain tumor patients could be further elaborated by the development of disease-specific questionnaires and using long-term (longitudinal) follow-up.

It would be desirable for researchers to also focus on nonmedical aspects in future studies, given their proven value.
